# What can mathematical modelling say about CHO metabolism and protein glycosylation?

**DOI:** 10.1016/j.csbj.2017.01.005

**Published:** 2017-01-28

**Authors:** Sarah N. Galleguillos, David Ruckerbauer, Matthias P. Gerstl, Nicole Borth, Michael Hanscho, Jürgen Zanghellini

**Affiliations:** a Department of Biotechnology, University of Natural Resources and Life Sciences, Vienna, Austria; b Austrian Centre of Industrial Biotechnology, Vienna, Austria

**Keywords:** 00-01, 99-00, CHO cells, Metabolic modelling, Glycosylation, MFA, Kinetic model, ^13^C-labelling, CHO, Chinese hamster ovary, FBA, Flux Balance Analysis, GSMR, genome-scale metabolic reconstruction, mAb, monoclonal antibody, MFA, metabolic flux analysis, PPP, pentose phosphate pathway, TCA, tricarboxylic acid

## Abstract

Chinese hamster ovary cells have been in the spotlight for process optimization in recent years, due to being the major, long established cell factory for the production of recombinant proteins. A deep, quantitative understanding of CHO metabolism and mechanisms involved in protein glycosylation has proven to be attainable through the development of high throughput technologies. Here we review the most notable accomplishments in the field of modelling CHO metabolism and protein glycosylation.

## 1 Introduction

Mammalian cells, more specifically immortalized Chinese hamster ovary (CHO) cells, are the dominant biological platform for the production of many therapeutic recombinant proteins [1]. CHO cells are not only able to correctly fold these proteins, but they are also capable of performing human-compatible post-translational modifications (e.g. glycosylation) [2], [3]. This is important for the correct functioning of the proteins and to prevent immunogenic responses in humans. In addition, CHO cells show high and stable expression of heterologous proteins and they easily adapt to growth in suspension. Both features are essential for industrial-scale production [4]. Furthermore, CHO cells are considered to be “safe”, since most human pathogenic viruses do not replicate in CHO [5]. All of these characteristics have contributed to a steep increase in the number of approvals for products expressed in this system compared to those produced in non-mammalian cells [6].

Due to their major role in the biopharmaceutical industry, several efforts have been focused on optimizing the culture process [7], [8]. In the past two decades, these efforts were mainly based on experimental observations of the metabolic profiles during cell culture [9], [10]. However, the advent of -omics technologies and associated modelling approaches facilitated a better and more detailed understanding of cell behaviour and intercellular processes. In particular, the development of constraint-based modelling techniques contributed tremendously to our understanding of metabolic processes, pathways and networks, so that these techniques have become one of the most (if not the most) successful modelling approaches in systems biology. Key to this success is the analysis of genome-scale metabolic reconstructions (GSMR). Combined with constraint-based modelling approaches, these models provide a mechanistic basis to investigate and elucidate genotype-phenotype relationships [11], [12].

Here we will review recent progress in the computational modelling of CHO cells. Specifically, we will focus on and analyze two main issues associated with recombinant protein production: (i) metabolic burdens affecting growth and thus protein yield and (ii) understanding of the correct glycosylation process of the protein of interest, which is one of the major criteria for product quality.

## 2 CHO metabolism

The cultivation of CHO cells in bio-reactors is characterized by fast consumption of the main carbon and energy sources, glucose and glutamine, with the concomitant production of ammonia and lactate. The production of lactate not only indicates inefficient metabolisation of the carbon sources [two molecules of ATP compared to 36 if glucose was completely oxidized in the tricarboxylic acid (TCA) cycle], but also has a negative effect on pH and osmolarity [13], which reduces the specific growth rate [14], [15] and protein yield [16]. High ammonia concentration in the medium has similar adverse effects on cell growth, productivity and glycosylation [17], [18], [19], [20]. Several strategies have been devised to overcome the accumulation of these by-products: rational supplementation of glucose and glutamine in fed-batch cultures [21], [22], use of alternative carbon sources [7] or cell engineering [23], [24], among others. These approaches were, however, based on trial and error and lack deterministic, quantitative justification.

### 2.1 Modelling CHO metabolism

To gain mechanistic understanding of these processes, appropriate metabolic models are required that allow one to estimate cellular flux distributions. This can be done in two ways: (i) in a time-dependent or dynamic manner (kinetic analysis) or (ii) in a constraint-based, steady-state analysis. The former approach aims to assess the evolution of the concentrations of metabolites over time and requires a large number of kinetic parameters. Due to the lack of accurate, quantitative data, this approach is currently not feasible on a genome-scale level, but restricted to small-scale models that consider several tens of selected reactions and interactions. The latter approach, on the other hand, avoids the need for detailed kinetic information by focusing on the steady-state behaviour inside the cell. Disregarding dynamic processes makes this approach, called metabolic flux analysis (MFA), scalable and suitable for genome-wide analysis. For better understanding the modelling approaches are briefly reviewed in Box 1.

**Fig. 1:**
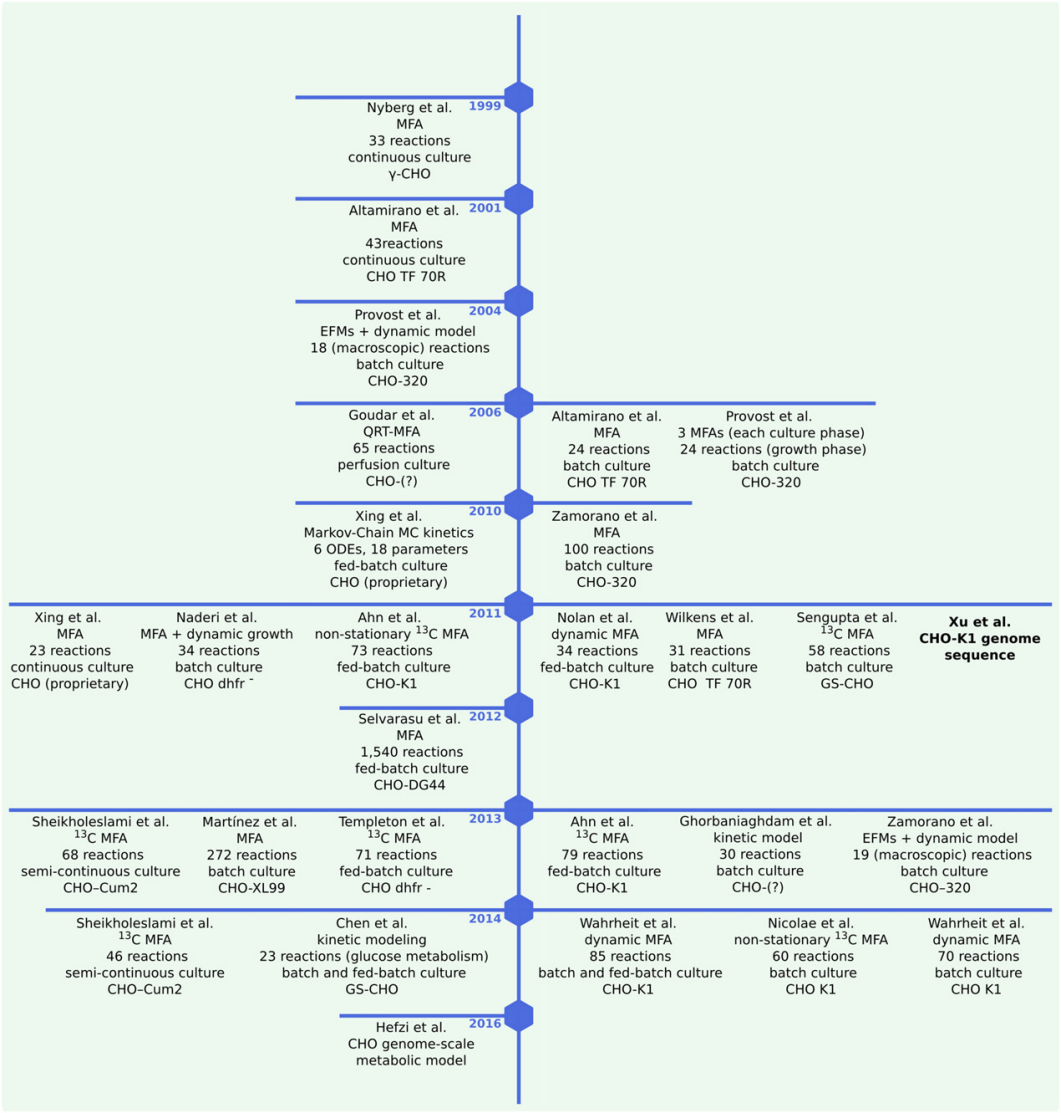
Metabolic modelling efforts in CHO listed in chronological order. Abbreviations: QRT, quasi-real-time; dhfr, dihydrofolate reductase.

In the following section we review current advances in metabolic modelling of CHO cells (listed chronologically in Fig. 1), focusing on those that investigate the accumulation of the two main metabolic by-products that are detrimental to cell growth, i.e. lactate and ammonia.

#### 2.1.1 The metabolic fate of lactate

Altamirano et al. [31] investigated the metabolic fate of lactate on a metabolic network of CHO core metabolism. They argued that, when re-metabolized, lactate is not used as an energy source, as their experimentally measured low oxygen uptake rate was inconsistent with a full oxidation of lactate via the TCA cycle. Consequently, they proposed alternative pathways for the non-oxidative decarboxylation of pyruvate, which are known to exist in cancer cells [32], to be present in CHO cells too. Nevertheless, the accumulation of the end product of these pathways, i.e. acetoin, was not experimentally proven. In a more recent work, Martinez et al. [33] were able to refute this hypothesis. In their study, they analyzed the metabolic switch from lactate production to lactate uptake by means of FBA in a reduced mouse-derived metabolic model. Contrary to Altamirano et al., Martinez et al. showed that their oxygen uptake rate measurements were consistent with lactate oxidation in the TCA cycle. This suggests that the metabolic network of Altamirano et al. might have been too simplistic to capture the metabolic changes between the phases. Compared to Martinez, Altamirano's model lacked fatty acid, steroid and glycogen metabolism. In addition, the prediction of the ATP yield per mol carbon identified lactate consumption to be energetically more efficient than glucose consumption. Furthermore, they showed that the estimation of ranges for the metabolic fluxes (due to the insufficient amount of experimentally measured data in an underdetermined network) provides a valuable, semi-quantitative description of the changes between the two metabolic states. This concept was also supported by Zamorano et al. [34], who performed MFA in an under-determined network containing 100 reactions of the core metabolism and obtained narrow intervals for the fluxes with a relatively low amount of extracellular measurements.

FBA can be combined with isotopomer analysis to improve the accuracy of the predicted fluxes. Sengupta et al. [35] studied the main metabolic fluxes in a simplified network during the stationary phase of cell culture by ^13^C MFA. This phase is typically characterized by reduced production of lactate and high protein yields. Likewise, Templeton et al. [36] performed ^13^C MFA to understand the metabolic changes between growth and stationary phases in a producer CHO cell line. They found that, during the antibody production peak (stationary phase), fluxes through the TCA cycle were maximal while lactate was not produced. Moreover, this increased activity of the TCA cycle correlated with increased fluxes through the oxidative pentose phosphate pathway (PPP) when compared to the exponential phase, where high glycolytic fluxes predominate. They provide several explanations for the activation of the oxidative PPP: to regenerate NADPH/NADP^+^, to compensate reduction during exponential growth, to suppress oxidative stress or to cover NADPH requirements during protein folding and secretion. Irrespective of the ultimate reason, these findings point towards metabolic engineering to increase oxidative TCA cycle (CO_2_-producing reactions) and PPP fluxes which would help achieve higher protein yields.

#### 2.1.2 Lactate as a beneficial medium component?

More recently, Chen et al. [37] even suggested that adding small amounts of lactate at the beginning of the culture process increases the metabolic efficiency. They used a kinetic model of the central carbon metabolism (i.e. glycolysis, PPP and TCA cycle) coupled with a model of the population dynamics and computed the time-dependent yield of lactate with respect to glucose. They found this yield decreased with increasing (yet not toxic) initial extracellular concentrations of lactate, meaning more efficient use of glucose. These findings were supported by Li et al. [38], who found that lactate can be fed as a major carbon source when glucose concentrations are kept low in culture.

Lactate uptake in the presence of galactose was also studied by flux balance analysis (FBA) in tissue plasminogen activator producing CHO cells in batch cultures [39]. Main changes were observed to occur in the pyruvate metabolism; the slow utilization of galactose as compared to glucose does not provide enough pyruvate to fulfill the energy requirements. This causes lactate dehydrogenase to reverse its mode of operation, transforming lactate into pyruvate, which then enters the TCA cycle. Consequently, intracellular pyruvate and lactate concentrations are reduced, which activates the monocarboxylate transporter towards lactate uptake.

The importance of taking compartments into consideration when modelling metabolism has been demonstrated by analyzing enzyme localized activity together with non-stationary ^13^C techniques. These allow a more accurate assessment of metabolic fluxes [40], mostly for those pathways that cannot be resolved using steady state approaches, such as cyclic or parallel pathways (e.g. glycolysis and PPP). In this study, Nicolae et al. also discussed the sources of lactate production in both cytosol and mitochondria. Taking into account not only the time-evolution of the metabolites, but also their spatial localization, proved that there is an additional control factor of precursor availability for both glycolysis and TCA cycle [41].

Likewise, Ahn et al. [42], [43] performed high precision ^13^C MFA on a network containing 79 reactions and resolved metabolic fluxes accurately. During the exponential phase, characterized predominantly by high fluxes through glycolysis, 70% of the glucose was converted to lactate. They also observed a decrease in glycolytic fluxes and an increase in the oxidative PPP in the stationary phase, as reported previously [36].

#### 2.1.3 What makes a “good” growth medium?

As already mentioned, the addition of alternative energy feedstocks can reduce the accumulation of undesired by-products. The effects of these alternative carbon sources on metabolism and protein production were studied with MFA on a reduced metabolic network by Altamirano et al. [44]. They showed that replacing glutamine by glutamate indeed resulted in reduced accumulation of ammonia, although at the price of a lower glucose uptake rate. This lowered metabolism has a negative impact on the specific protein production rate, as carbon is predominantly captured to sustain growth, leaving little for protein production.

In a follow-up work, Altamirano et al. [31] considered co-feeding strategies with galactose added to the medium, as galactose-glutamate media are known to significantly reduce by-product formation, but unfortunately, also cell growth. However, they showed that after glucose depletion, cells were able to maintain growth on galactose by simultaneously utilizing previously produced lactate. Interestingly, CHO cells do not metabolize lactate when it is offered as the sole carbon source.

MFA has also been applied for media optimization. Xing et al. performed MFA in continuous culture to assess the metabolic demands (in terms of amino acids) of antibody producing CHO cells [45], which resulted in a modified medium where final concentrations of ammonia and lactate were reduced and higher viable cell densities and higher productivities were achieved.

The steady state assumption might be problematic when modelling the inherently time-dependent fed-batch processes [46]. Hence, several efforts have been made to perform kinetic metabolic analysis while keeping a reduced, tractable set of reactions to avoid dealing with too many kinetic parameters. One of the first attempts in this direction was made by Nolan et al. [47], who included kinetic expressions in a reduced, lumped model containing 34 reactions. They studied the metabolic lactate switch by linking glucose concentration in the medium to cytosolic levels of NADH and lactate metabolic rate (lower levels of cytosolic NADH leading to net lactate consumption). This study also analyzed the intracellular concentrations of 24 metabolites in different cell lines and found that 20 of them either remained constant during the process or that their concentration changes were negligible compared to the fluxes, supporting the validity of the pseudo-steady state assumption [25] also for fed-batch processes.

Goudar et al. [48] made remarkable progress towards quasi real-time estimation of the metabolic rates in perfusion culture of CHO cells for optimal process control based on metabolite balancing. They observed that reducing the initial concentrations of glucose and glutamine resulted in an increased flux towards the TCA cycle and decreased production of waste metabolites, mainly lactate.

Xing et al. [49] applied a Markov chain Monte Carlo method to develop a kinetic model of fed-batch cultures and predicted optimal initial concentrations of glucose and glutamine that minimized the production of ammonia and lactate.

The effects of decreasing concentrations of glutamine in the media, namely the increased uptake of other carbon sources and the reduction of secreted ammonia and other products, was studied by dynamic MFA on fed-batch CHO cultures with different glutamine concentrations [50]. They show how controlled feeding prevents glutamine metabolism to be coupled to waste producing pathways and, moreover, stabilizes the flux through the TCA cycle.

Similarly, Sheikholeslami et al. [51] used ^13^C MFA to compare two semicontinuous cultures grown on chemically defined media with 1 mM and 5 mM glutamine, respectively, and found that low glutamine uptake (in the 1 mM culture) was more metabolically efficient in terms of the proportion of pyruvate that enters the TCA cycle (and therefore is not converted to lactate). Furthermore, the CHO cell line used in this study was found to be particularly efficient, mostly under hypothermic conditions, as confirmed on their previous work [52]. In this case, the use of ^13^C MFA was simplified by analyzing only extracellular ^13^C -labelled metabolites and then performing MFA to predict the intracellular fluxes.

Another interesting feeding strategy was suggested by Naderi et al. [53]. In their work, they used MFA to reduce the metabolic network to a set of significant reactions and coupled them to a dynamic cell growth model to asses the differences between growing and apoptotic cells. They highlighted the differences on the metabolic rates for the different cell subpopulations (growing, resting and apoptotic cells) and suggested a feeding strategy based on the “aging” of the cell culture: when glutamine is in excess in late phases of the process (where the non-growing cells become predominant), there is a switch from glycolytic reactions towards deamination of glutamine (and concomitant ammonia accumulation), which could be prevented by gradually lowering the concentrations of glutamine in the feed as the culture ages.

Some other compounds, such as sodium butyrate, have shown to improve productivity in CHO cells [54]; Ghorbaniaghdam et al. [55] used a kinetic model to assess the effects of this compound on metabolism in a non-compartmentalized model assuming Michaelis-Menten kinetics. They found cells to become more energetically efficient (in terms of the lactate to glucose ratio) when sodium butyrate was added at the mid-exponential phase. Moreover, they made noteworthy improvements in describing energy metabolism (in terms of ATP) and redox potential (in terms of NADH, NAD^+^, NADPH and NADP^+^). Adding sodium butyrate to the media generates an increased flux through the TCA cycle and a high cell redox potential, while not significantly changing the ATP production rates.

MFA has also been combined with statistical analysis methods (such as principal component analysis) to determine key metabolites linked to the accumulation of ammonia and lactate. In their study, Selvarasu et al. [56] analyzed profiles of extracellular and intracellular species and integrated this information in a mouse-derived GSMR with the goal of finding pathways related to growth limitation. In addition to glucose and glutamine, they identified asparagine to be correlated with the accumulation of ammonia in the medium, most probably via its conversion to aspartate, then glutamate and finally *α*-ketoglutarate.

#### 2.1.4 The future starts now: *i*CHO1766, a comprehensive, genome-scale metabolic reconstruction of CHO

As outlined above, the results derived from a model-based analysis have significantly improved our understanding of the underlying metabolic processes. This is all the more remarkable as, so far, a truly CHO-specific GSMR was missing. All the applications summarized above used either small-scale metabolic models or adapted reconstructions developed for related organisms like mouse or humans. However, after the complete genomic sequence of CHO-K1 was published in 2011 [57], several research groups around the world joined forces in creating the first community-curated GSMR of CHO, which just now became available [58]. This model consists of 4455 metabolites participating in 6663 reactions and contains 1766 annotated genes. In a first demonstration of possible applications of this CHO GSMR, typical process engineering strategies were analyzed for their effects on the predicted maximum product yield. In all tested cases, the model suggested that these processes are not even close to tapping the full potential of CHO cells.

Furthermore, the transcriptome [59] and proteome [60] of CHO cells can be now used to obtain strain-specific models that provide a more precise characterization of metabolic capabilities [61]. Metabolomics data can further refine these models to make better predictions under the given culture conditions. Thus, given the advances in high-throughput technology, we expect that the model based-analysis of systems-level data like the transcriptome and proteome will help to further unravel the complexity of CHO metabolism.

Regardless of these promising results, model performance has to be further evaluated. Ever since the first modelling approaches appeared, the accuracy of experimental measurements has been shown to be an important factor to obtain meaningful results [62]. Moreover, it has been shown that biomass composition varies among different cell lines [56]. It is also known that the biomass composition has a great effect on model predictions [63]. Therefore, factors influencing the robustness of CHO metabolic models is a question that still remains to be addressed.

## 3 Glycosylation

Modelling metabolism aims at reducing the metabolic burden on the cells induced by the recombinant production of the protein of interest. It aims to increase the protein yield. However, the biopharmaceutical industry is not only faced with the problem of producing therapeutic proteins efficiently, but also to produce them at high quality. A major quality attribute of many biopharmaceuticals is correct glycosylation, as the correct function of most therapeutic proteins depends on it [64]. Glycosylation consists of the addition of an oligosaccharide chain to an amino acid residue, predominantly asparagine (N-linked) or serine/threonine (O-linked glycosylation) and takes place in the endoplasmic reticulum and Golgi apparatus along the protein secretory pathway. These sugar modifications play a fundamental role in protein conformation, stability, solubility, receptor recognition and antigenicity as well as cytotoxicity [65], [66], [67], [68]. Thus glycosylation essentially modifies the pharmacological properties of a protein.

Glycosylation patterns are naturally and in general heterogeneous. There are two main sources of variability in glycosylation: macroheterogeneity, which refers to the fact that a particular site in the protein might or might not be glycosylated; and microheterogeneity, when different glycan structures can be found on the same site. However, this natural variability presents a particular challenge for the production of biosimilars, were the glycosylation patterns of the primary drugs have to be reproduced within tight tolerance regions defined by regulatory authorities [117].

### 3.1 Modelling glycosylation in CHO

Many factors are known to influence glycosylation in cell culture: concentration of metabolites in the medium (both substrate and waste products), pH, temperature and cell viability [69], [70]. The mechanisms by which these factors affect micro- and macroheterogeneity remain, however, unclear. Thus a systematic analysis is called for. Computational modelling provides a powerful framework for such an analysis. In fact, there have been remarkable advances in the development of mathematical models of glycosylation, supported by the detailed knowledge of the glycosylation pathways [71]. Generally, these models aim to reduce the combinatorial explosion in the number of possible glycan distributions. To this end, models make some general assumptions, while keeping compartmentalization (each compartment is modelled differently since they contain different sets of enzymes) and finally linking glycosylation to metabolism. The complexity of the process, together with the many intervening factors, makes modelling glycosylation quite a challenging task.

One of the first attempts to deterministically describe protein glycosylation focused on macroheterogeneity. In 1996, Shelikoff et al. [72] proposed a mathematical model to predict how site-occupancy is affected by different factors such as the expression levels of glycotransferases, the protein production rate, the concentrations of nucleotide sugars and the mRNA elongation rate. They used a plug-flow reactor-based model and included protein folding as a competing event that occurs concurrently with glycosylation.

Shortly after, Monica et al. [73] modeled sialylation of N-linked oligosaccharides in a single, isotropic compartment (trans-Golgi). The predictions were in agreement with experimental data of CD4 glycoprotein produced in CHO cells.

Umaña and Bailey (1997) [74] presented the first attempt to model glycoform microheterogeneity based on expression and spatial localization of the enzymes involved in N-linked glycosylation. Parameters such as the half-life of the protein in the Golgi, the protein productivity and the volume of the Golgi compartments were also included in this model. Furthermore, they modified the model to take the competition for the glycosylation machinery between endogenous and recombinant proteins into account. Kontoravdi et al. used this model of glycosylation and included it in a simple dynamic mathematical model of cell growth, death and metabolism. With this reduced model they predicted the evolution of oligosaccharide molar fractions over time. However, these results could not be validated due to the lack of experimental data [75].

Several years later, in 2005, Krambeck and Betenbaugh [76] extended Umaña's model (which contained 33 glycan structures and 33 reactions), by adding around 7500 oligosaccharide structures and more than 22,000 reactions. Among these, reactions for fucosylation and sialylation were included in the model, which are of special relevance for recombinant proteins [77], [78]. In contrast to the model of Umaña and Bailey, this model adjusts enzyme concentrations to fit an experimentally observed glycopattern, thereby calibrating it to a specific protein. They argue that the reason for having a case-specific, adjusted model is the inherent variability of glycosylation: the glycan structures do not only depend on the specific protein, but also on the glycosylation site. Their results were validated with N-glycan structures observed in recombinant human thrombopoietin expressed in CHO cells [79]. This model was then used as a prototype for further development by other research groups.

In 2009, Krambeck et al. applied the previously developed model to predict enzyme expression that resulted in an observed mass spectrometry spectrum. Reciprocally, the model was used to automatically annotate spectra to the corresponding glycan structures [80].

Both models (Umaña and Bailey, Krambeck and Betenbaugh) were combined in two different studies to predict the sensitivity of N-Glycan branching with respect to the hexosamine flux [81] and key enzymes involved in glycan branching [82].

Senger and Karim [83] used a plug-flow reactor model to describe the differences in glycosylation of recombinant tissue plasminogen activator in CHO under shear stress conditions. They found decreased site occupancy to be related to low residence times of the protein in the endoplasmic reticulum due to high protein production rates, caused by increasing levels of shear stress.

In a follow-up study, Senger and Karim used artificial neural network models to predict glycosylation from primary sequence information around the glycosylation site (glycosylation window). The model was used to classify macroheterogeneity as either robust (invariant with culture conditions) or variable, according to this sequence information [84]. They improved this approach further by using information about the secondary structure and solvent accessibility, resulting in the prediction of two main types of glycan branching: high mannose type and complex-type [85]. Artificial neural networks had already been applied to predict glycosylation sites [86], [87]. The complexity of the impact of protein conformation in the surroundings of the glycosylation site on glycotransferase activity hinders the creation of a mathematical model that could describe the process deterministically. Therefore, they presented the Neural-Network approach as a valuable workaround to construct prediction tools. The main advantage of this approach with respect to the previous models is that it does not require a large number of parameters, but only the protein sequence (from which they predict the secondary structure). In addition, it highlights the influence of protein secondary and tertiary structure on the accessibility of the enzymes. In another instance, Gerken et al. [88] considered the inhibitory effect of the presence of glycan structures on neighboring sites of glycosylation.

Built on the premise that glycan biosynthesis is controlled by the expression of glycotransferases, Kawano et al. [89] predicted a set of glycan structures from DNA microarray data. This set was further expanded by Suga et al. [90] with the prediction of new structures (Kawano's set of predicted glycans was limited to those included in the database of known structures). This approach was refined several years later with high-throughput RNA microarray data [91].

Hossler et al. [92] compared the prediction performance of two main models for protein maturation in the Golgi: four continuous mixing-tanks for vesicular transport and four plug-flow reactors in series for the maturation model. They claimed that the latter describes the process more accurately and they emphasised the importance of the residence time in the Golgi and enzyme localization as key parameters to be considered when modelling glycosylation.

The plug-flow reactor model was then used to describe monoclonal antibody (mAb) glycosylation [93]. The major improvement over the previous model was to include the transport of nucleotide sugar donors. This was the first step towards coupling cellular metabolism (and therefore measurable variables like glucose uptake) to glycosylation. Kaveh et al. [94] pursued this goal and performed a dynamic analysis of extracellular metabolite concentrations via MFA and linked those of glutamine and glucose to nucleotide sugar biosynthesis and glycolysis using the previous models (del Val 2011 [93] and Hossler 2007 [92]). The model successfully predicted dynamic trends of the glycopatterns of mAb produced in CHO batch culture. In another study [95], they combined dynamic MFA with the GLYCOVIS software developed by Hossler et al. [96] to predict, based on experimentally observed glycopatterns, how different concentrations of glutamine, glucose, ammonia and different pH values affect the glycosylation process. Yet more progress was made by Jedrzejewski et al. [97], who used a dynamic model for cell death and growth together with the dynamic model from del Val [93] to predict glycosylation patterns. In this case, experimental data from mAb producing mouse hybridoma cells was used for the calculations. A similar study was applied to mAb producing CHO fed-batch cultures [98]. As a result, recent models have succeeded in linking cell growth, metabolism, protein production rate and glycosylation [99].

The majority of these models describe N-glycosylation. Liu et al. [100] presented a reaction network for the formation of the O-glycosylation of the sialyl Lewis-X epitope. In their work, they introduce the concept of “subset-modelling”, where the whole set of reactions in the network is divided into “sub-networks” and then a search is performed for the one that fits the experimental data best. Furthermore, they use genetic algorithm-based optimization, hierarchical clustering and principal component analysis to fit subsets of reaction networks to the observed glycan structure distribution, thereby reducing the parameterisation of the model. Recently, the same group developed a software for the automated creation, analysis and visualization of glycosylation reaction networks, called GNAT (Glycosylation Network Analysis Toolbox) [101], [102]. GNAT was further expanded to include a higher number of enzymes [103].

Kim et al. [104] also exploited the modularity of the glycosylation pathways to propose new engineering strategies based on targeting modules instead of specific enzymes.

In a simpler approach, FBA was applied to assess the effect of low temperature conditions on metabolism and nucleotide sugar availability for glycosylation in mAb producing CHO cells [105]. A similar MFA-based method was applied to analyze the effects of different concentrations of glutamine in the media on nucleotide sugar intracellular concentrations and N-glycan content of recombinant human chorionic gonadotrophin in CHO cells [106].

In the past year, a simple stoichiometric model was also used to compute the nucleotide sugar demands for glycosylation of recombinant proteins in CHO for rational feeding strategies [107].

In order to avoid the requirement of a high number of kinetic parameters, Spahn et al. [108] used a Markov chain model to describe glycosylation as a stochastic process in which each glycan state has a transition probability to reach the next glycan state. These probabilities are linked to the steady state solution given by FBA for a reduced network of the reactions contributing to the observed glycoprofile. By using this protein-specific model, they successfully predicted the effect of an enzyme knock-down on an antibody producer CHO cell line [109].

### 3.2 Parameters and general assumptions

The parameters involved in glycosylation include reaction kinetic parameters, compartment residence times, enzyme distributions between compartments, compartment volumes, total glycan concentration and donor cosubstrate concentrations. These parameters are either obtained via optimization or taken from literature [110]. Imaging techniques for green fluorescent protein-labelled proteins can be used to measure residence time and protein flux through the secretory machinery [111]. Kinetic parameters are commonly derived from independent enzymology experiments [112], which are arduous and should be carried out for each enzyme. However, there have been remarkable advances on high-throughput technologies that allow more accurate assessment of kinetic parameters of glycosyltransferases [113].

Due to the sequential nature of glycosylation, models have to incorporate time-dependent equations. The majority of the kinetic models reviewed herein assume Michaelis-Menten Kinetics. Over time, more terms were included in these models' equations, with increasing complexity, e.g. competitive inhibition terms in their enzyme-kinetic expressions.

The main limitation of glycosylation models is the high grade of parameterisation required to describe the process. Moreover, most of the parameters are derived from in vitro experiments, even though they might be different in an intracellular environment. As previously mentioned, various factors influence glycosylation at different points of the process [70] and the effects are cell line [114], glycoprotein [115] and even glycosylation site specific [74], which reduces the general applicability of the models. Thus, despite the tremendous advances achieved over the last years in this field, the ultimate goal of predicting the effect of cell line specific behaviour of different protein sequences or structures, or of process related changes on glycosylation still requires further work and ptimisation to be fully achieved.

## 4 Conclusions and future perspectives

Metabolic modelling of mammalian cells has been hampered by the inherent complexity of the cell structure (compartmentalization) and the large variability of media compositions and process perturbations under which the culture processes are carried out. Nevertheless, with the rampant progress in scope and reliability of -omics technologies, it is for the first time that we can access cell metabolism in a systems-level manner. The main applications are the rational improvement of both the culture process (media optimization) and the cells themselves (via targeted genetic engineering).

The natural evolution towards more complex metabolic models including compartmentalization and dynamic analysis has put emphasis on the necessity to have accurate measurements(intra- and extracellular) as well as accurate values for the biomass and media composition [56], [116]. As for glycosylation, it has been recently shown that only a limited amount of CHO proteins account for the majority of glycosylation, which could ease the approaches dealing with the dynamic evolution of glycosylation by focusing solely on these highly contributing proteins [107].

To date, the vast majority of modelling approaches in CHO have been applied in a reduced set of reactions. These usually include glycolysis, TCA cycle, PPP and amino acid metabolism. However, in the past year, a full genome-scale metabolic model of CHO has become available, unleashing the capabilities of genome-scale metabolic modelling.

**Fig. 2:**
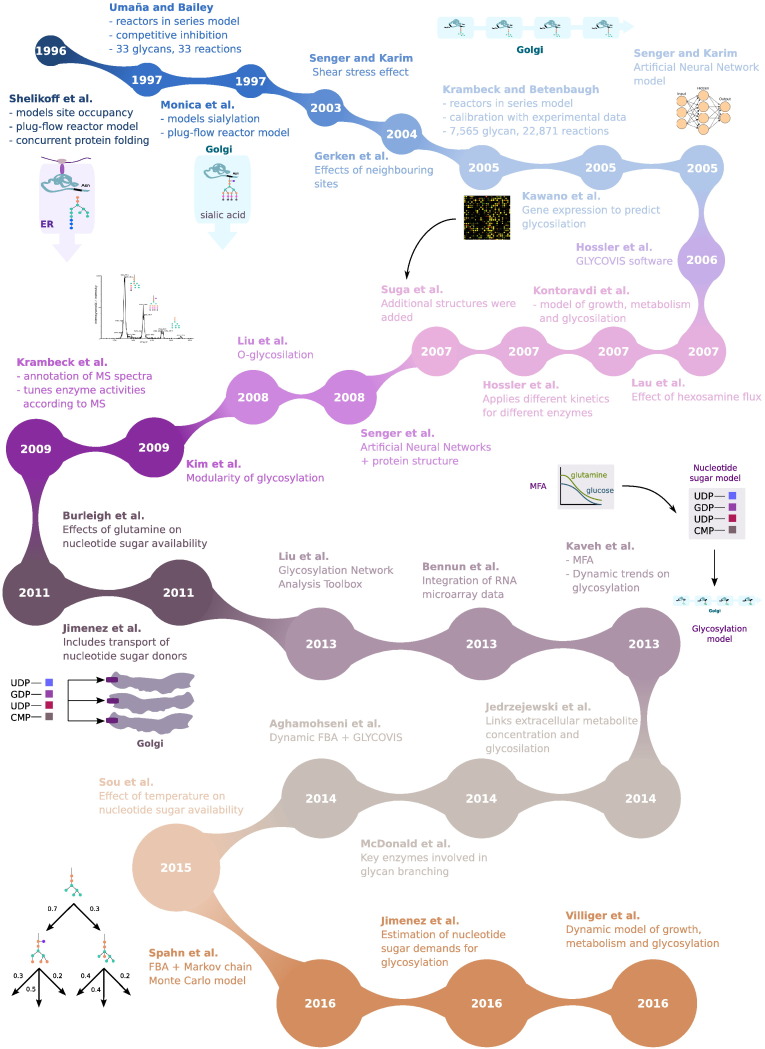
Models for protein glycosylation in CHO listed in chronological order. Abbreviations: MS, Mass-spectrometry.

We have also addressed the second main challenge concerning the production of recombinant proteins in CHO. Glycosylation is a highly complex, variable process, in which many factors are involved. Glycan patterns vary from batch to batch and from strain to strain, making it difficult to model the process deterministically. Even though the mechanisms by which the culture conditions and enzyme expression affect glycosylation are still unknown, the modelling efforts discussed here (see Fig. 2) have taken a significant step forward in media optimization by linking glycosylation to metabolism. A future step in this direction would be including glycan compounds in the biomass stoichiometric equation, since it has been shown that the metabolic demands towards glycosylation of both recombinant and host proteins are significant [107].

Therefore, given the combinatorial nature of the process, there are still major achievements to be reached in controlling the glycoform, since it plays a key role in product quality.

## Box 1 Common modelling approaches.

**Table:**

**MFA** (Metabolic Flux Analysis): pathway analysis method based on the stoichiometry of metabolic reactions and mass balances under pseudo-steady-state assumption [25]. It can be implemented in several ways. Among them: • **FBA** (Flux Balance Analysis): an implementation of MFA based on the optimization of a cellular function (such as growth) under specific constraints (experimental metabolic uptake and secretion rates, thermodynamic data, etc.) [26], [27]. • **^13^C MFA**: isotope-labelled substrates are added to the culture media and, once the isotopic steady-state is reached, the distribution of the isotopes is measured via nuclear magnetic resonance or gas chromatography—mass spectrometry [28]. **Markov chain Monte Carlo** sampling: the glycosylation process is described as a series of states with transition probabilities from one state to the other. In the references reviewed herein, it is used to overcome the lack of kinetic parameters (metabolic and glycosylation enzymes) [29]. **Artificial Neural Network models**: aim to predict the behaviour of complex, non-linear systems by detecting and “learning” patterns and relationships within a training set which can be applied then to the input data [30].

Alt-text: Box 1
